# Turkish cultural adaptation and psychometric evaluation of the children activity scales–parent and teacher forms (ChAS-P/T)

**DOI:** 10.3389/fped.2026.1906645

**Published:** 2026-08-21

**Authors:** Duygu Demirbas, Merve Cebi, Teresa A. May-Benson, Aymen Balikci

**Affiliations:** 1 Faculty of Humanities and Social Sciences, Department of Psychology, Üsküdar University, İstanbul, Türkiye; 2 Faculty of Arts and Sciences, Department of Psychology, Mimar Sinan Fine Arts University, Istanbul, Türkiye; 3 TMB Education, Norristown, PA, United States; 4 Sense On Ltd., Istanbul, Türkiye

**Keywords:** children activity scales, cross-cultural adaptation, developmental coordination disorder, psychometric properties, validity and reliability

## Abstract

**Background:**

Developmental Coordination Disorder (DCD) is a prevalent neurodevelopmental condition that negatively affects children's participation in daily activities at home and school. Screening tools based on parent and teacher reports are particularly valuable for early identification, as they capture functional performance in natural contexts. The Children Activity Scales–Parent and Teacher Forms (ChAS-P/T) were developed for this purpose; however, a Turkish version for Turkish children has not previously been available.

**Objective:**

This study aimed to translate and culturally adapt the ChAS-P/T into Turkish and to examine its reliability and validity in children aged 4–8 years.

**Methods:**

The study followed internationally recommended guidelines for cross-cultural adaptation and psychometric validation. The ChAS-P/T was translated, culturally adapted, and pre-tested before psychometric evaluation. Parents and teachers of 340 Turkish children aged 4–8 years, including typically developing children and 34 children diagnosed with DCD, participated. The psychometric evaluation assessed the reliability, construct validity, discriminant validity, and criterion-related validity of the Turkish version.

**Results:**

The Turkish ChAS-P and ChAS-T demonstrated excellent internal consistency and strong test–retest reliability. Confirmatory factor analyses supported the original four-factor structure of the parent form and three-factor structure of the teacher form. Significant group differences supported discriminant validity. ROC analyses indicated good to excellent criterion-related validity, with optimal cut-off scores yielding high sensitivity and specificity. Strong correlations between parent and teacher reports further supported convergent validity across informants.

**Conclusions:**

The Turkish versions of the ChAS-P and ChAS-T are reliable and valid screening instruments for identifying children aged 4–8 years at risk for DCD. Their robust psychometric properties and multi-informant structure support their use in clinical and educational settings to facilitate early identification and referral.

## 1 Introduction

Developmental Coordination Disorder (DCD) is a persistent neurodevelopmental condition that markedly impairs an individual's capacity to acquire and execute fundamental motor skills necessary for daily activities and academic functions (1). Children with DCD struggle to acquire and refine new motor abilities, as well as to perform previously acquired motor skills efficiently. Consequently, they may experience difficulties in motor-based activities, including sports or games requiring catching, jumping, or running, school-related tasks such as writing or using scissors, and activities of daily living, including dressing, brushing teeth, and tying shoes (2–7). DCD is estimated to affect approximately 5%–6% of children (3, 4, 6). Despite its relatively high prevalence and well-established diagnostic criteria, DCD remains inadequately recognized by key stakeholders, including parents, educators, and healthcare professionals (5).

The Diagnostic and Statistical Manual of Mental Disorders (DSM-5) defines DCD as a neurodevelopmental disorder characterized by significant motor coordination difficulties that emerge during early development, interfere with activities of daily living, academic achievement, and participation, and cannot be better explained by intellectual disability, visual impairment, or other neurological conditions (3). Although the DSM-5 provides clear diagnostic criteria, international clinical recommendations emphasize that DCD should be identified through a comprehensive multidisciplinary assessment, including developmental and medical history, interviews, clinical examination, standardized motor assessments, and questionnaires. Furthermore, assessment should evaluate children's performance in activities of daily living, participation in recreational activities, academic functioning, and the consistency between performance in standardized testing and natural environments (4). In line with these recommendations, several parent- and teacher-reported questionnaires have been developed to screen children for probable DCD and to capture the impact of motor difficulties on daily participation. Commonly used instruments include the Developmental Coordination Disorder Questionnaire [DCDQ'07; (8)], a parent-report screening questionnaire for identifying children at risk for DCD; the Movement Assessment Battery for Children-2 Checklist [MABC-2 Checklist; (9)], an observational checklist of motor performance in everyday settings; the Motor Observation Questionnaire for Teachers [MOQ-T; (10)], a teacher-report measure of motor coordination difficulties; the DCDDaily-Q (11), which evaluates children's performance in activities of daily living; and the Children Activity Scales–Parent and Teacher Forms [ChAS-P/T; (12)], which assess functional performance in home and school environments. A comprehensive review of DCD-related questionnaires has highlighted these instruments as commonly used screening tools (13, 14). While these questionnaires differ in their structure, target age range, and psychometric properties, none is considered sufficient as a standalone diagnostic tool; rather, they constitute important components of a comprehensive assessment process (4, 14). Among these tools, the Children Activity Scales–Parent/Teacher (ChAS-P/T) were developed by Rosenblum (12) to identify children aged 4–8 years at risk for DCD by evaluating their performance in everyday activities within natural contexts, such as home and school. The instrument consists of parallel parent and teacher questionnaires that provide complementary perspectives on children's functional performance across different environments. Unlike many other screening questionnaires, the ChAS-P/T places a strong emphasis on activities of daily living, participation, and the organization of movement in time and space, domains that are central to the DSM-5 diagnostic criteria for DCD yet are often underrepresented in other screening instruments (12, 13). The ChAS-P/T has demonstrated high internal consistency and strong specificity, supporting its usefulness for the early identification and referral of children at risk for DCD, particularly during the preschool years when motor difficulties may first become apparent (12, 14). To the best of our knowledge, no published cross-cultural adaptation or psychometric validation studies of the ChAS-P/T in languages other than the original version are currently available.

In the Turkish context, the DCDQ'07 has been translated, culturally adapted, and demonstrated to be a valid and reliable screening instrument for children aged 5–15 years (15). However, the DCDQ'07 was developed primarily for school-aged children and focuses mainly on general coordination, fine motor skills, and control during movement. In contrast, the ChAS-P/T was specifically designed for younger children aged 4–8 years and provides a broader assessment of functional performance across multiple domains, including fine and gross motor skills, activities of daily living, self-care, mobility, ball skills, play, and common school or preschool activities within children's natural environments. By incorporating reports from both parents and teachers and emphasizing children's everyday functioning, the ChAS-P/T offers greater ecological validity and complements the existing Turkish DCDQ'07 rather than replacing it. Consequently, there is currently no culturally adapted Turkish screening instrument specifically designed to evaluate functional performance and participation in young children aged 4–8 years at risk for DCD using this comprehensive, participation-focused approach.

Therefore, the purpose of this study was to translate and culturally adapt the ChAS-P/T into Turkish and to evaluate its psychometric properties. As the first published cross-cultural adaptation and psychometric evaluation of the ChAS-P/T, this study aims to provide a valid and reliable screening instrument for identifying Turkish children aged 4–8 years at risk for DCD. Specifically, this study sought to determine whether the Turkish version of the ChAS-P/T is a reliable and valid instrument for identifying children aged 4–8 years at risk for DCD by examining its reliability, construct validity, discriminant validity, and diagnostic accuracy. In addition, the study investigated whether the Turkish version of the ChAS-P/T could serve as a culturally appropriate screening instrument for use with Turkish children. It is expected to facilitate early identification and referral for comprehensive assessment and timely intervention, thereby addressing an important gap in the Turkish assessment literature and supporting international recommendations for early identification and participation-focused evaluation (4, 12). Beyond providing a culturally adapted screening instrument, this study contributes to pediatric occupational therapy by supporting participation-focused and occupation-based assessment of young children with or at risk for DCD.

## 2 Materials and methods

The study followed internationally recommended procedures for the cross-cultural adaptation and psychometric evaluation of assessment instruments (16–19). Prior to data collection, formal permission to use and adapt the Children Activity Scales–Parent and Teacher Forms (ChAS-P/T) was obtained from the original scale developer. Ethical approval was obtained from the Üsküdar University Non-Interventional Research Ethics Committee (Approval No. 61351342/October 2022–33; approval date: 28 October 2022). The study was conducted in accordance with the ethical principles of the Declaration of Helsinki.

The study was conducted in three sequential phases. Part 1 involved the translation and cultural adaptation of the ChAS-P/T into Turkish. Part 2 focused on evaluating reliability, including internal consistency (Cronbach's alpha and item–total correlations) and test–retest reliability using intraclass correlation coefficients (ICCs). Part 3 examined validity, including construct validity through confirmatory factor analysis, discriminant validity by comparing typically developing children with children diagnosed with DCD, and criterion-related validity using receiver operating characteristic (ROC) curve analyses to determine optimal cut-off scores.

Typically developing children were recruited from public kindergartens and primary schools located in Istanbul. School principals and teachers were contacted by telephone and were provided with detailed information about the purpose and procedures of the study before institutional permission was obtained. Children with DCD who had been diagnosed by a child psychiatrist and referred for occupational therapy were recruited from two private occupational therapy clinics in Istanbul. Data were collected between November 2022 and April 2023.

Written informed consent was obtained from the parents or legal guardians of all participating children and from all participating teachers before data collection. The questionnaires were administered during face-to-face sessions, and participants required approximately 5–10 min to complete the instruments.

### 2.1 Instruments

The ChAS-P/T are screening questionnaires designed to identify children aged 4–8 years who may be at risk for DCD by evaluating their performance in everyday activities within natural home and school environments. The scales emphasize functional performance rather than isolated motor skills and assess areas that are central to the diagnostic criteria for DCD, including activities of daily living, fine and gross motor skills, and the organization of movement in time and space (12).

The ChAS-P/T were originally developed in Hebrew by Rosenblum (12). An English version of the scale was subsequently made available by the original author as a standardized translation. The present Turkish translation and cultural adaptation were conducted using this English version obtained directly from the scale developer. Both the parent and teacher forms are brief, practical screening tools that take approximately 8–10 min to complete.

The ChAS-parent form (ChAS-P) consists of 27 items distributed across four domains: fine motor skills (5 items), gross motor skills (6 items), organization in space and time (6 items), and activities of daily living (9 items). Respondents are asked to compare the child's performance with that of same-aged peers using a 5-point Likert scale, ranging from “very well” (5) to “less adequately” (1), with higher scores indicating better performance. In the original standardization study, the parent form showed high internal consistency (Cronbach's *α* = 0.94). The mean total score was 4.39 (SD = 0.57), and a cut-off score of 3.82 (one standard deviation below the mean) was established to identify children suspected of having DCD (12).

The ChAS-teacher form (ChAS-T) was developed for teachers working with children aged 4–8 years and includes 21 items derived from the parent form. Items related to activities of daily living were removed from the teacher version because teachers reported difficulty observing these behaviors in the school context. Consequently, the ChAS-T assesses three domains: fine motor skills, gross motor skills, and organization in space and time. The original study reported excellent internal consistency for the teacher form (Cronbach's *α* = 0.96). Based on data from 355 teachers, a cut-off score of 3.42 (mean minus one standard deviation) was established to classify children at risk for DCD (12).

Evidence supporting the psychometric properties of the ChAS-P/T includes strong internal consistency, a clear, theoretically coherent factor structure, and the ability of both forms to discriminate between typically developing children and those diagnosed with DCD. Additionally, concurrent validity has been demonstrated through significant correlations with performance-based motor assessments, particularly the M-ABC, while maintaining high specificity, supporting the use of the ChAS-P/T as effective screening tools rather than diagnostic instruments (12, 14).

### 2.2 Procedures and participants

The study was conducted in accordance with internationally recognized recommendations for the translation, cross-cultural adaptation, and psychometric validation of health-related measurement instruments (16, 18–20). The translation and cultural adaptation process comprised seven consecutive stages prior to psychometric evaluation: forward translation, synthesis of the translations, linguistic review, back-translation, comparative review, expert cognitive interviews, and pre-testing. Throughout this process, semantic, idiomatic, experiential, and conceptual equivalence between the original and Turkish versions of the ChAS-P/T was systematically evaluated to ensure linguistic accuracy, cultural relevance, and conceptual consistency. Different participant groups, including translators, language experts, healthcare professionals, parents, teachers, and children, contributed to the respective stages of the adaptation process. The procedures undertaken and the characteristics of the participants involved at each stage are presented in Table 1.

**Table 1:** Participants involved at each stage of the study.

Study phase	Step	Participant group	Qualifications/characteristics	N
Translation & cultural adaptation	Step 1—Forward Translation	Translator 1 (F1)	Graduate of English Language and Literature; >5 years’ experience in health-related translations	1
		Translator 2 (F2)	Professional translator/interpreter; >15 years’ experience in medical and rehabilitation translations	1
	Step 2—Synthesis	Synthesis Team	Clinical psychologist (PhD, >10 years clinical experience); pediatric physiotherapist (PhD, >15 years combined experience); occupational therapist (>5 years pediatric clinical experience)	3
	Step 3—Linguistic Evaluation	Language Experts	PhD in special education (>15 years preschool experience); PhD in Turkish language and literature (>20 years academic experience)	2
	Step 4—Back Translation	Medical Translators	Professional medical translators with >10 years’ experience in rehabilitation related translations.	2
	Step 5—Review	Synthesis Team	Review of semantic, conceptual, and contextual equivalence	3
	Step 6—Expert Interview	Clinicians	Occupational therapists and physiotherapists with >5 years pediatric clinical experience.	4
	Step 7-Pre-Testing	Parents and Teachers	Parents and teachers of typically developing children aged 4–8 years; evaluation of the clarity, comprehensibility, cultural appropriateness, and feasibility of the Turkish version.	30
Psychometric evaluation	Step 8	Typically Developing Children	Children aged 4–8 years attending public kindergartens and primary schools in Istanbul.	340
		Children with DCD	Children aged 4–8 years diagnosed with DCD by a child psychiatrist or neurologist and referred for occupational therapy.	34

Clinicians involved in Step 6 were independent experts recruited from pediatric rehabilitation centers and were not part of the earlier translation or synthesis teams. The number of 4-year-old participants in the main validation sample was relatively lower compared to other age groups and this imbalance was considered in the interpretation of findings.

The translation and cultural adaptation procedures were implemented in seven consecutive stages, each designed to ensure the linguistic and cultural equivalence of the Turkish version:

Step 1 (Forward Translation): Two bilingual translators independently translated the original ChAS-P/T into Turkish.
Step 2 (Synthesis): The two forward translations were synthesized into a single preliminary Turkish version by a multidisciplinary committee comprising a clinical psychologist, a pediatric physiotherapist, and an occupational therapist. Any discrepancies were resolved through discussion until consensus was achieved.
Step 3 (Linguistic Review): The synthesized version was evaluated by Turkish language experts to assess grammatical accuracy, comprehensibility, and cultural appropriateness. Revisions were made where necessary while preserving the conceptual meaning of the original items.
Step 4 (Back-Translation): The revised Turkish version was independently back-translated into English by professional medical translators who were blinded to the original questionnaire.
Step 5 (Comparative Review): The original questionnaire and the back-translated version were systematically compared by the research team to identify discrepancies and verify semantic, idiomatic, experiential, and conceptual equivalence. Any inconsistencies were resolved by consensus, resulting in the pre-final Turkish version.
Step 6 (Expert Cognitive Interviews): The pre-final Turkish version was evaluated by experienced clinicians through structured cognitive interviews. The experts assessed the clarity, comprehensibility, relevance, cultural appropriateness, and usability of the questionnaire and provided recommendations for improving wording and item interpretation where required.
Step 7 (Pre-testing): The revised pre-final version was administered to the parents and teachers of 30 typically developing children aged 4–8 years. Following questionnaire completion, structured cognitive interviews were conducted to evaluate the clarity, comprehensibility, feasibility, and acceptability of the questionnaire within the target population. Feedback obtained during this stage was reviewed by the research team, and minor revisions were incorporated into the final Turkish version before psychometric evaluation.

Following completion of the translation and cultural adaptation process, the psychometric properties of the Turkish version of the ChAS-P/T were evaluated. The sample size was considered adequate for psychometric evaluation based on current methodological recommendations for questionnaire validation studies. Although no universal consensus exists regarding the minimum sample size required for psychometric studies, larger samples are recommended because they improve the stability of factor structures, reduce measurement error, increase the accuracy of parameter estimates, and enhance the generalizability of findings (21–23). Furthermore, recent psychometric guidelines emphasize that sample size adequacy should be considered in relation to the study design, factor analytic approach, and measurement objectives rather than relying on a single numerical criterion (24). Therefore, the total sample of 374 children included in the present study was considered sufficient to support the confirmatory factor analysis and other psychometric evaluations performed.

The questionnaire was administered to the parents and teachers of 340 typically developing children and 34 children diagnosed with developmental coordination disorder (DCD), aged 4–8 years. The collected data were used to examine internal consistency, test–retest reliability, construct validity, discriminant validity, and the diagnostic accuracy of the Turkish ChAS-P/T.

To evaluate temporal stability, the ChAS-P/T was re-administered to the parents and teachers of a subsample of 33 children after a three-week interval. This interval is commonly recommended in psychometric research because it minimizes recall bias while reducing the likelihood of true changes in the construct being measured (25). Internal consistency was assessed using Cronbach's alpha coefficients, test–retest reliability using intraclass correlation coefficients (ICCs), construct validity through confirmatory factor analysis (CFA), discriminant validity by comparing the scores of typically developing children and children with DCD, and diagnostic accuracy using receiver operating characteristic (ROC) curve analysis by calculating the area under the curve (AUC), optimal cut-off scores, sensitivity, and specificity (25).

### 2.3 Data analysis

All statistical analyses were performed using IBM SPSS Statistics for Windows, Version 22.0 (26) and IBM SPSS AMOS, Version 24.0 (IBM Corp., Armonk, NY, USA). Descriptive statistics (frequencies, percentages, means, and standard deviations) were calculated using SPSS. Reliability analyses included internal consistency (Cronbach's alpha and item–total correlations), test–retest reliability assessed with intraclass correlation coefficients (ICC), and short-term score consistency evaluated using dependent-samples *t*-tests.

Validity analyses were conducted using both SPSS and AMOS. Construct validity was examined through a confirmatory factor analysis (CFA) in AMOS, selected for its specialization in structural equation modeling and latent variable estimation (27, 28). Model fit was evaluated using the chi-square to degrees of freedom ratio (*χ*^2^/*df*), Goodness-of-Fit Index (GFI), Adjusted Goodness-of-Fit Index (AGFI), Comparative Fit Index (CFI), Root Mean Square Error of Approximation (RMSEA), and Root Mean Square Residual (RMR) (29). Discriminant validity was assessed using independent-samples *t*-tests comparing typically developing children and children diagnosed with Developmental Coordination Disorder (DCD), while criterion-related validity was examined using receiver operating characteristic (ROC) curve analyses in SPSS to determine the area under the curve (AUC), optimal cut-off scores, sensitivity, and specificity for the parent and teacher forms.

## 3 Results

### 3.1 Descriptive characteristics of the psychometric evaluation sample

The study sample consisted of 374 children aged 4–8 years (mean age = 6.14 ± 1.36 years), including 340 (90.9%) typically developing children and 34 (9.1%) children diagnosed with DCD. The sample included 168 boys (44.9%) and 206 girls (55.1%). Most parents were married (77.8%), while 22.2% were divorced. The majority of parents had completed a university or postgraduate education (65.0%), whereas 35.0% had completed primary or high school education. More than half of the parents were unemployed (57.2%) at the time of data collection. Overall, the demographic characteristics indicate a heterogeneous sample with respect to family structure, parental education and employment status, and child characteristics, supporting the representativeness of the study sample (see Table 2).

**Table 2:** Descriptive characteristics of the psychometric evaluation sample.

Variable	Category	Frequency (n)	Percentage (%)
Diagnostic status	Typically developing	340	90.9
	Diagnosed with DCD	34	9.1
Parental marital status	Married	291	77.8
	Divorced	83	22.2
Parental education level	Primary school	11	2.9
	High school	120	32.1
	University degree	209	55.9
	Postgraduate degree	34	9.1
Parental employment status	Employed	160	42.8
	Unemployed	214	57.2
Child's gender	Male	168	44.9
	Female	206	55.1
Child's age (years)	4	50	13.3
	5	87	23.3
	6	74	19.8
	7	87	23.3
	8	76	20.3

Total sample size: *N* = 374. Percentages are calculated within each variable.

### 3.2 Translation and cultural adaptation

The translation and cultural adaptation process demonstrated a high level of agreement across all stages. Both forward translators (100%) reported that the original English version was easy to translate into Turkish without major linguistic difficulties. Following independent translation, the multidisciplinary synthesis team compared the two versions item by item and reached unanimous agreement (100%) that both translations were conceptually equivalent and easy to understand. A single consensus Turkish version was subsequently developed by selecting the most appropriate wording from the two forward translations.

The synthesized version was evaluated by two language experts, both of whom (100%) agreed that the Turkish version was grammatically correct, clear, and comprehensible. Minor recommendations, including punctuation refinements and the addition of brief explanatory expressions to improve clarity, were incorporated into the revised version.

Comparison of the back-translated and original English versions showed no conceptual inconsistencies. Although minor syntactic differences were identified, all members of the synthesis team (100%) agreed that these reflected natural linguistic adaptation rather than changes in meaning and that the conceptual integrity of the original instrument had been fully preserved.

During expert interview, all four occupational therapists and physiotherapists (100%) rated both the parent and teacher forms as easy to understand, clearly worded, and clinically appropriate. All experts reported that the items could be interpreted without difficulty and considered the forms suitable for routine clinical use.

In the pre-testing phase, the finalized Turkish version was completed by the parents and teachers of 30 children aged 4–8 years. Cognitive interviews indicated that all participants (100%) considered the items to be clear, easy to understand, and culturally appropriate. No participant reported problems related to wording, ambiguity, cultural relevance, or item interpretation.

### 3.3 Construct validity

**Figure 1:**
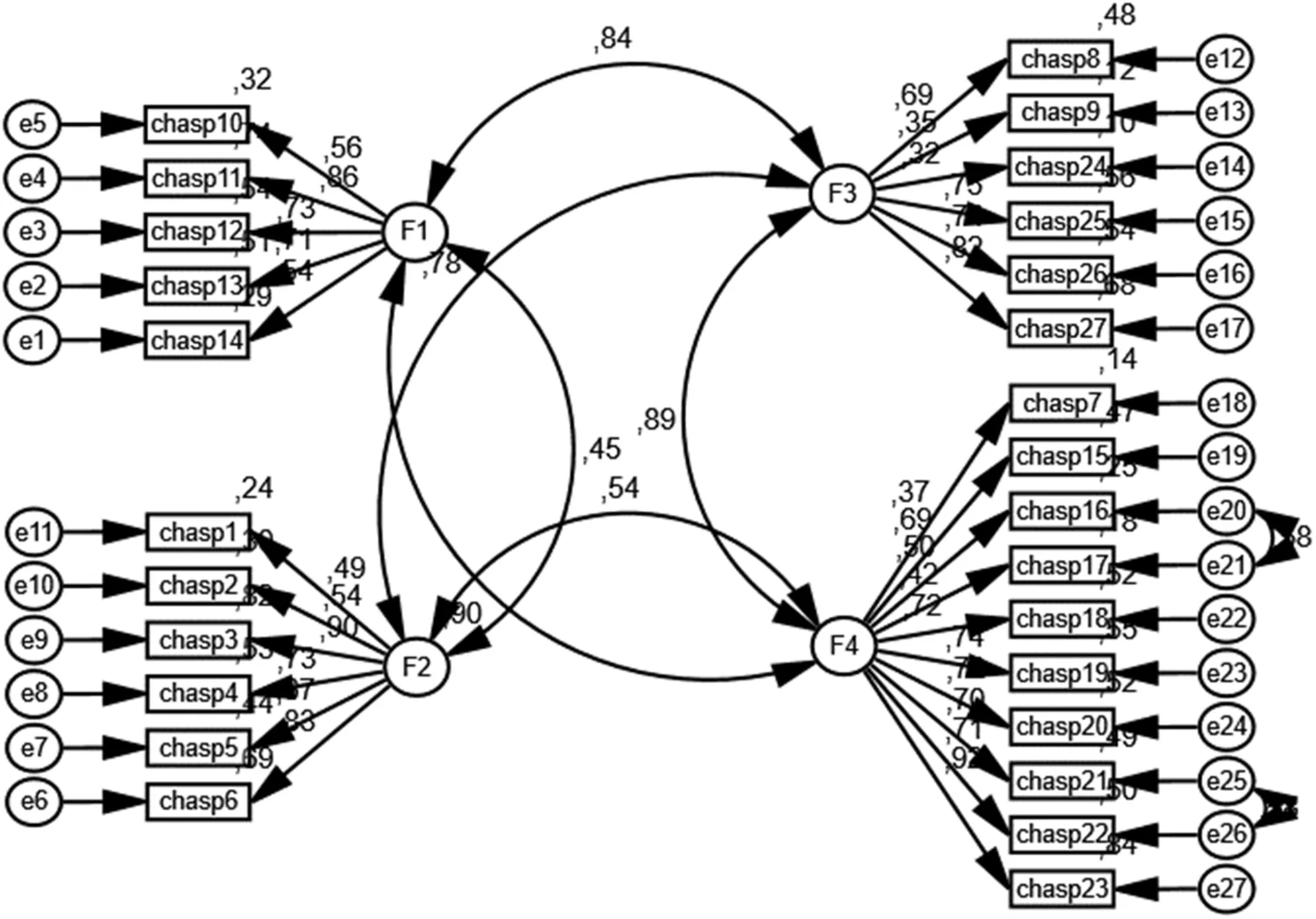
Diagram for confirmatory factor analysis of ChAS-P.

**Figure 2:**
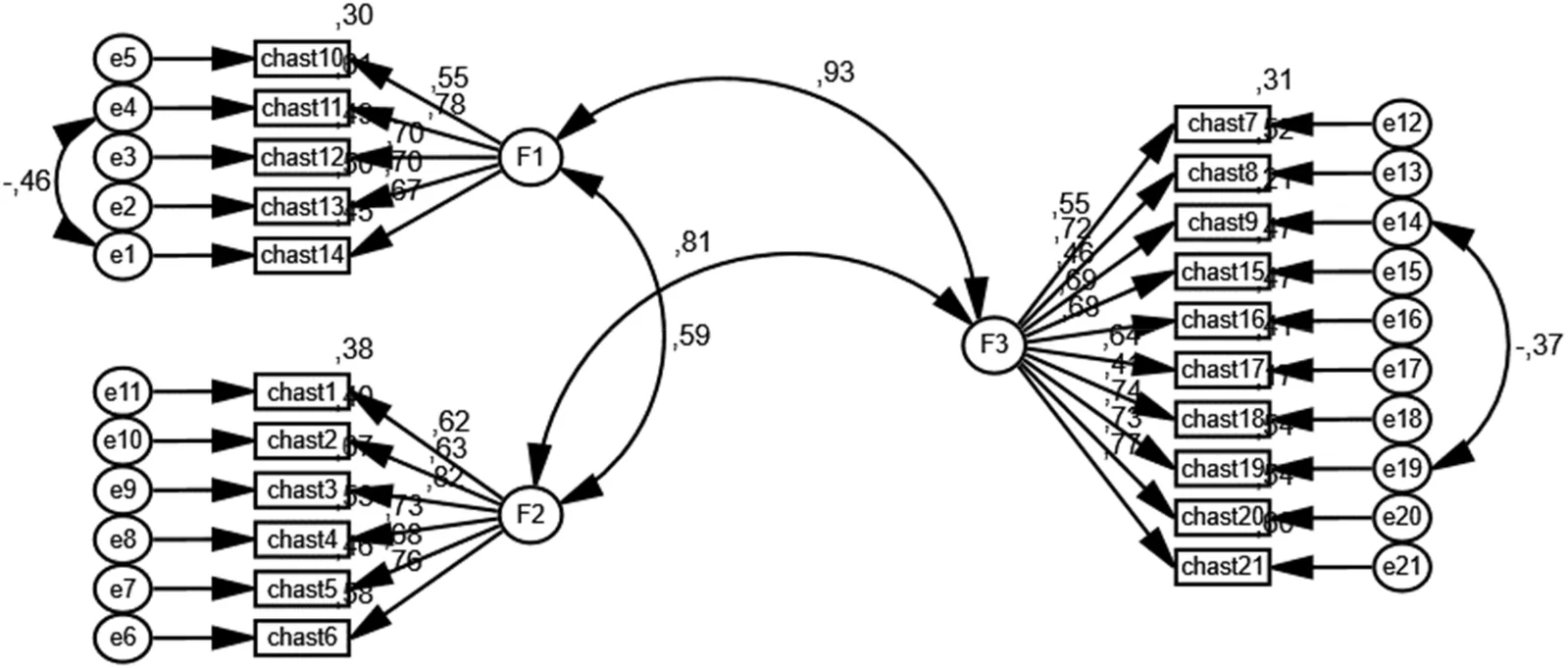
Diagram for confirmatory factor analysis of ChAS-T.

Confirmatory factor analysis demonstrated an acceptable model fit for both the ChAS-P and ChAS-T. For the ChAS-P, the fit indices were *χ*^2^/*df* = 2.63, GFI = 0.90, AGFI = 0.90, CFI = 0.93, RMSEA = 0.06, and RMR = 0.07, indicating that the Turkish parent form adequately fit the original factor structure (Table 3, Figure 1). Similarly, the ChAS-T demonstrated an acceptable model fit (*χ*^2^/*df* = 3.48, GFI = 0.90, AGFI = 0.90, CFI = 0.91, RMSEA = 0.06, and RMR = 0.07), supporting the original factor structure of the teacher form (Table 4, Figure 2).

**Table 3:** ChAS-P confirmatory factor analysis Index values.

Index	Normal value	Acceptable value	Value
*χ*^2^/sd	<2	<5	2.63
GFI	>0.95	>0.90	0.90
AGFI	>0.95	>0.90	0.90
CFI	>0.95	>0.90	0.93
RMSEA	<0.05	<0.08	0.06
RMR	<0.05	<0.08	0.07

*χ*^2^/*df*, chi-square to degrees of freedom ratio; GFI, Goodness-of-Fit Index; AGFI, Adjusted Goodness-of-Fit Index; CFI, Comparative Fit Index; RMSEA, Root Mean Square Error of Approximation; RMR, Root Mean Square Residual.

**Table 4:** ChAS-T confirmatory factor analysis Index values.

Index	Normal value	Acceptable value	Value
χ2/sd	<2	<5	3.48
GFI	>0.95	>0.90	0.90
AGFI	>0.95	>0.90	0.90
CFI	>0.95	>0.90	0.91
RMSEA	<0.05	<0.08	0.06
RMR	<0.05	<0.08	0.07

*χ*^2^/*df*, chi-square to degrees of freedom ratio; GFI, Goodness-of-Fit Index; AGFI, Adjusted Goodness-of-Fit Index; CFI, Comparative Fit Index; RMSEA, Root Mean Square Error of Approximation; RMR, Root Mean Square Residual.

### 3.4 Reliability of the Turkish versions of the ChAS-P/T

Reliability analyses demonstrated excellent internal consistency for the Turkish version of the ChAS-P. The Cronbach's alpha coefficient for the total scale was 0.938, indicating a high level of internal consistency. Item–total correlation coefficients ranged from 0.381 to 0.755, showing that all items contributed meaningfully to the overall construct. Item-deletion analyses revealed that removal of any item did not result in an improvement in the overall alpha coefficient, supporting retention of all items (see Table 5).

**Table 5:** Item analysis of the Turkish ChAS-P.

Item	Scale score when item is deleted	Variance when item is deleted	Item–total correlation	Cronbach's alpha when item is deleted
Item 1	106.42	234.834	0.388	0.937
Item 2	106.20	231.307	0.447	0.937
Item 3	106.39	226.072	0.628	0.935
Item 4	106.33	229.224	0.419	0.938
Item 5	106.56	226.279	0.521	0.936
Item 6	106.35	227.584	0.641	0.935
Item 7	106.52	227.521	0.490	0.937
Item 8	106.40	223.453	0.689	0.934
Item 9	106.20	231.637	0.381	0.938
Item 10	106.08	232.478	0.440	0.937
Item 11	106.48	219.071	0.755	0.933
Item 12	106.28	227.558	0.606	0.935
Item 13	106.65	220.592	0.600	0.935
Item 14	106.45	227.728	0.575	0.936
Item 15	106.68	225.966	0.678	0.935
Item 16	106.59	226.832	0.578	0.936
Item 17	106.68	227.210	0.455	0.937
Item 18	106.82	221.164	0.555	0.936
Item 19	106.84	219.245	0.692	0.934
Item 20	106.63	224.669	0.636	0.935
Item 21	107.04	221.869	0.634	0.935
Item 22	106.88	222.206	0.634	0.935
Item 23	106.96	216.888	0.822	0.932
Item 24	106.79	233.279	**0**.**285**	0.939
Item 25	106.74	222.332	0.675	0.934
Item 26	106.57	223.591	0.700	0.934
Item 27	106.90	217.456	0.782	0.933

Test–retest reliability analyses were conducted with forms corresponding to 33 children. For the parent form, the intraclass correlation coefficient for the total score indicated excellent temporal stability (ICC = 0.912). Paired-samples *t*-test results revealed no statistically significant difference between test and retest administrations [*t*(32) = −0.158, *p* = 0.876] (see Table 6).

**Table 6:** Test–retest reliability and intraclass correlation coefficients for the Turkish ChAS-P (*n* = 33).

Measure	Test Mean ± SD	Retest Mean ± SD	*t*	*p*	ICC	*p*
Total Score	4.093 ± 0.494	4.094 ± 0.488	−0.158	.876	0.912	<.001
Fine Motor Skills	4.388 ± 0.485	4.382 ± 0.483	0.442	.662	0.906	<.001
Gross Motor Skills	4.202 ± 0.608	4.177 ± 0.576	1.305	.201	0.936	<.001
Organization in Space and Time	4.101 ± 0.629	4.086 ± 0.624	1.359	.184	0.922	<.001
Activities of Daily Living	3.876 ± 0.506	3.906 ± 0.506	1.398	.158	0.917	<.001

Test–retest reliability was evaluated using paired-samples *t*-tests and intraclass correlation coefficients (ICC). ICC, Intraclass Correlation Coefficient.

Similarly, the teacher form demonstrated strong temporal stability, with a high intraclass correlation coefficient for the total score (ICC = 0.923). No significant difference was observed between test and retest scores [*t*(32) = −1.000, *p* = 0.325], further supporting score stability over time (see Table 7). The Turkish version of the ChAS–T also demonstrated high internal consistency, with a Cronbach's alpha coefficient of 0.929. Item–total correlation coefficients ranged from 0.402 to 0.739, and no items fell below the acceptable threshold. Item-deletion analyses supported retention of all items (see Table 8).

**Table 7:** Item analysis of the Turkish ChAS-T.

Item	Scale score when item is deleted	Variance when item is deleted	Item–total correlation	Cronbach's alpha when item is deleted
Item 1	81.99	157.791	0.550	0.927
Item 2	81.78	156.288	0.552	0.926
Item 3	81.98	152.951	0.649	0.925
Item 4	81.96	152.902	0.543	0.927
Item 5	82.11	152.288	0.600	0.926
Item 6	81.96	155.132	0.624	0.925
Item 7	82.07	154.512	0.549	0.926
Item 8	82.01	150.592	0.709	0.923
Item 9	81.84	158.028	0.420	0.929
Item 10	81.77	157.857	0.465	0.928
Item 11	82.07	151.073	0.653	0.924
Item 12	81.93	154.279	0.626	0.925
Item 13	82.25	149.458	0.615	0.925
Item 14	82.04	154.859	0.593	0.926
Item 15	82.24	153.290	0.655	0.925
Item 16	82.37	150.190	0.646	0.925
Item 17	82.42	152.271	0.581	0.926
Item 18	82.40	156.696	0.402	0.930
Item 19	82.34	149.635	0.703	0.923
Item 20	82.16	151.279	0.704	0.924
Item 21	82.44	147.443	0.739	0.923

**Table 8:** Test–retest reliability and intraclass correlation coefficients for the Turkish ChAS-T (*n* = 33).

Measure	Test Mean ± SD	Retest Mean ± SD	*t*	*p*	ICC	*p*
Total Score	4.211 ± 0.440	4.218 ± 0.435	−1.000	.325	0.923	<.001
Fine Motor Skills	4.412 ± 0.482	4.418 ± 0.483	−0.442	.662	0.899	<.001
Gross Motor Skills	4.222 ± 0.527	4.232 ± 0.517	−0.812	.423	0.908	<.001
Organization in Space and Time	4.103 ± 0.477	4.109 ± 0.473	−0.702	.488	0.919	<.001

Test–retest reliability was evaluated using paired-samples *t*-tests and intraclass correlation coefficients (ICC). ICC, Intraclass Correlation Coefficient.

### 3.5 Criterion-related and discriminant validity

Receiver operating characteristic (ROC) curve analysis demonstrated good criterion-related validity for both forms of the Turkish ChAS-P/T. For the ChAS-P, the area under the curve (AUC) was 0.842 (95% CI = 0.801–0.877, *p* < .0001), with an optimal cut-off score of ≤3.81, yielding a sensitivity of 88.24% and specificity of 77.94% (see Table 9). For the ChAS-T, the AUC was 0.972 (95% CI = 0.950–0.986, *p* < .0001), with an optimal cut-off score of ≤3.50, resulting in sensitivity and specificity values of 94.12% (see Table 10).

**Table 9:** Significance of the Roc curve in relation to the Turkish ChAS-P.

Parameter	Value
Area under the Roc curve (AUC)	**0** **.** **842**
Standard error	0.024
95% confidence interval	0.801–0.877
*Z* statistic	14.536
*p*-Area = 0.5	<0.0001
Youden index J	0.662
Cut off	**≤3.81**
Sensitivity	88.24
Specificity	77.94

Bold values indicate the area under the receiver operating characteristic curve (AUC).

**Table 10:** Significance of the Roc curve in relation to Turkish ChAS-T.

Parameter	Value
Area under the Roc curve (AUC)	**0** **.** **972**
Standard error	0.008
95% confidence interval	0.950–0.986
Z statistic	55.972
*P* (Area = 0.5)	<0.0001
Youden index J	0.882
Cut off	**≤3.50**
Sensitivity	94.12
Specificity	94.12

Bold values indicate the area under the receiver operating characteristic curve (AUC).

Discriminant validity was evaluated by comparing the scores of typically developing children and children diagnosed with DCD using independent-samples *t*-tests. For the parent form, typically developing children (*n* = 340) obtained significantly higher total scores than children with DCD (*n* = 34) [4.153 ± 0.564 vs. 3.558 ± 0.399; *t*(372) = 6.001, *p* < .001]. Significant differences were also observed across all subscales, including Fine Motor Skills [4.354 ± 0.660 vs. 3.553 ± 0.462; *t*(372) = 6.905, *p* < .001], Gross Motor Skills [4.370 ± 0.590 vs. 3.578 ± 0.477; *t*(372) = 7.578, *p* < .001], Organization in Space and Time [4.113 ± 0.619 vs. 3.628 ± 0.641; *t*(372) = 4.349, *p* < .001], and Activities of Daily Living [3.947 ± 0.693 vs. 3.506 ± 0.494; *t*(372) = 3.616, *p* < .001] (see Table 11). Similarly, for the teacher form, typically developing children (*n* = 340) obtained significantly higher total scores than children with DCD (*n* = 34) [4.222 ± 0.502 vs. 2.936 ± 0.418; *t*(372) = 14.438, *p* < .001]. Significant differences were also observed across all subscales, including Fine Motor Skills [4.327 ± 0.586 vs. 2.882 ± 0.524; *t*(372) = 13.821, *p* < .001], Gross Motor Skills [4.363 ± 0.561 vs. 3.020 ± 0.573; *t*(372) = 13.285, *p* < .001], and Organization in Space and Time [4.085 ± 0.580 vs. 2.912 ± 0.637; *t*(372) = 11.144, *p* < .001], demonstrating good discriminative validity of the Turkish ChAS-T (see Table 12).

**Table 11:** Differences in ChAS-P scores according to diagnostic Status.

Variable	Typically developing (*n* = 340) Mean ± SD	Children with DCD (*n* = 34) Mean ± SD	*t*	*df*	*p*
ChAS-P total score	4.153 ± 0.564	3.558 ± 0.399	6.001	372	<0.001
Fine motor skills	4.354 ± 0.660	3.553 ± 0.462	6.905	372	<0.001
Gross motor skills	4.370 ± 0.590	3.578 ± 0.477	7.578	372	<0.001
Organization in space and time	4.113 ± 0.619	3.628 ± 0.641	4.349	372	<0.001
Activities of daily Living	3.947 ± 0.693	3.506 ± 0.494	3.616	372	<0.001

**Table 12:** Differences in ChAS-T scores according to diagnostic Status.

Variable	Typically developing (*n* = 340) Mean ± SD	Children with DCD (*n* = 34) Mean ± SD	*t*	*df*	*p*
ChAS-T total score	4.222 ± 0.502	2.936 ± 0.418	14.438	372	<0.001
Fine motor skills	4.327 ± 0.586	2.882 ± 0.524	13.821	372	<0.001
Gross motor skills	4.363 ± 0.561	3.020 ± 0.573	13.285	372	<0.001
Organization in space and time	4.085 ± 0.580	2.912 ± 0.637	11.144	372	<0.001

### 3.6 Relationship between parent and teacher forms

Pearson correlation analysis was conducted to examine the relationship between the parent and teacher forms of the Turkish ChAS-P/T. Strong and statistically significant positive correlations were observed between the total scores of the two forms (*r* = 0.899, *p* < .001). Similarly, significant positive correlations were found between corresponding subscales, including Fine Motor Skills (*r* = 0.903, *p* < .001), Gross Motor Skills (*r* = 0.925, *p* < .001), and Organization in Space and Time (*r* = 0.884, *p* < .001). Correlations between the total scores and non-corresponding subscales ranged from 0.399 to 0.908 and were all statistically significant (*p* < .001), indicating good agreement between parent and teacher ratings of children's activity performance (see Table 13).

**Table 13:** Correlation analysis between the Turkish ChAS-P and ChAS-T scores.

ChAS-T score	Statistic	ChAS-P total Score	Fine motor skills	Gross motor skills	Organization in space and time	Activities of daily living
ChAS-T total score	r	0.899**	0.772**	0.759**	0.839**	0.774**
	*p*	<0.001	<0.001	<0.001	<0.001	<0.001
Fine motor skills	r	0.779**	0.903**	0.402**	0.688**	0.713**
	*p*	<0.001	<0.001	<0.001	<0.001	<0.001
Gross motor skills	r	0.676**	0.399**	0.925**	0.602**	0.495**
	*p*	<0.001	<0.001	<0.001	<0.001	<0.001
Organization in space and time	r	0.908**	0.766**	0.687**	0.884**	0.811**
	*p*	<0.001	<0.001	<0.001	<0.001	<0.001

ChAS-P, children activity scale–parent; ChAS-T, children activity scale–teacher.

Pearson correlation analysis.

** Indicates a statistically significant correlation at *p* < .01.

## 4 Discussion

The present study aimed to translate and culturally adapt the ChAS-P and ChAS-T into Turkish and to evaluate their psychometric properties in a sample of Turkish children aged 4–8 years. Overall, the findings indicate that the Turkish versions demonstrate satisfactory linguistic and cultural equivalence with the original instrument, together with strong evidence of structural validity, reliability, and criterion-related validity (12). The rigorous cross-cultural adaptation process, supported by input from language experts, clinicians, parents, and teachers, ensured that the items were conceptually equivalent and culturally relevant for use in Turkish home and school settings. Furthermore, the findings from confirmatory factor analysis, internal consistency, test–retest reliability, discriminative validity, and criterion-related validity collectively demonstrate that the Turkish ChAS-P and ChAS-T are psychometrically sound screening instruments for identifying children at risk for developmental coordination disorder. These findings support the use of the Turkish ChAS-P and ChAS-T in both clinical practice and educational settings and address an important need for a culturally appropriate, standardized screening instrument for Turkish-speaking children. The cultural adaptation findings of the present study highlight the importance of a systematic, multi-informant approach when adapting participation-focused screening tools across languages and cultures (17, 18, 30, 31). Consistent with international recommendations, the adaptation process extended beyond literal translation by evaluating semantic equivalence, contextual relevance, and cultural appropriateness through expert review, cognitive interviews, and pre-testing (16–18, 32).

The relatively smooth forward translation and synthesis process may be attributed to both the expertise of the translators and the characteristics of the ChAS-P/T itself. The translation team included professionals experienced in medical and rehabilitation terminology, while the multidisciplinary synthesis committee integrated expertise in occupational therapy, pediatric rehabilitation, and clinical psychology. Furthermore, the ChAS-P/T consists of concise items describing familiar, observable everyday activities rather than abstract psychological constructs or culturally specific concepts. These characteristics likely facilitated consensus during synthesis and minimized the need for substantial revisions. Similar observations have been reported in other Turkish adaptation studies, in which forward translations demonstrated strong semantic consistency and required only minor modifications before consensus was achieved (20, 33).

Although several discrepancies emerged during the back-translation stage, these mainly reflected linguistic refinements and the addition of brief explanatory wording to improve clarity in Turkish rather than changes to the underlying concepts. Such differences are expected during rigorous cross-cultural adaptation because achieving conceptual equivalence often requires natural linguistic expressions rather than literal translation. Importantly, these modifications did not alter the intended meaning of the items, and similar findings have been reported in previous Turkish adaptation studies, where wording refinements improved comprehensibility while preserving conceptual equivalence (18, 20, 33).

The consistently positive evaluations provided by clinicians, parents, and teachers further support the cultural appropriateness and usability of the Turkish ChAS-P/T. This finding may be explained by the instrument's focus on functional behaviours that are routinely observed across home and school environments, enabling respondents to evaluate children's performance with relatively little ambiguity. Moreover, involving language experts, healthcare professionals, parents, and teachers throughout the adaptation process likely contributed to the identification and resolution of potentially ambiguous wording before psychometric evaluation. Consequently, no linguistic or cultural concerns were identified during cognitive interviews or pre-testing, suggesting that the Turkish version successfully preserved the conceptual intent of the original instrument while remaining readily understandable for its intended users. Similar findings have been reported in recent adaptation studies employing iterative expert review and end-user involvement (12, 18, 33).

Overall, these findings reinforce previous evidence that successful cross-cultural adaptation depends not only on accurate translation but also on systematic expert review and active involvement of end users to ensure semantic equivalence, cultural relevance, and practical usability in clinical settings (17–19, 31, 34). Similarly, previous psychometric validation studies conducted within the Turkish occupational therapy literature have reported that rigorous translation and cross-cultural adaptation procedures support the development of reliable and valid Turkish versions of standardized assessment instruments (35, 36).

Confirmatory factor analysis provided strong evidence for the structural validity of the Turkish versions of the ChAS-P and ChAS-T. The hypothesized four-factor structure of the parent form and three-factor structure of the teacher form were successfully replicated, demonstrating that the underlying conceptual organization of the original instrument was preserved following translation and cultural adaptation (12). Furthermore, the acceptable model fit indices (*χ*^2^/*df*, GFI, AGFI, CFI, RMSEA, and RMR) and significant standardized factor loadings indicate that the observed items adequately represent their intended latent constructs. These findings suggest that the Turkish ChAS-P/T maintains the theoretical framework of the original instrument while providing an appropriate representation of children's functional motor performance and participation within the Turkish cultural context.

The preservation of the original factor structure may be attributed to both the rigorous translation and cultural adaptation procedures and the nature of the ChAS-P/T itself. Unlike instruments assessing abstract psychological constructs, the ChAS-P/T evaluates observable functional performance and participation in familiar everyday activities, making the underlying constructs less susceptible to cultural variation and facilitating the maintenance of conceptual equivalence throughout the adaptation process (12). Similar findings have been reported in recent Turkish adaptation studies, where careful cross-cultural adaptation enabled translated instruments to retain their original conceptual framework while remaining culturally appropriate for the target population (20, 33).

This is particularly important in cross-cultural adaptation studies, as preservation of the original factor structure indicates that the adapted instrument measures the same underlying constructs across cultures, thereby supporting construct validity and cross-cultural equivalence (18, 37). Similar conclusions have been reported in previous cross-cultural validation studies, where replication of the original factor structure through confirmatory factor analysis was interpreted as evidence that the translated instrument retained its structural integrity and could be confidently applied in the target population (38, 39).

The reliability findings of the present study provide strong evidence for the consistency and stability of the Turkish versions of the ChAS-P and ChAS-T. Both forms demonstrated excellent internal consistency, with Cronbach's alpha coefficients exceeding commonly accepted thresholds, indicating that the items within each scale coherently measure the underlying construct of functional motor participation (40–42). These findings are highly consistent with those reported in the original development and standardization study of the ChAS-P/T, in which Rosenblum (12) reported Cronbach's alpha coefficients of.94 for the parent form and.96 for the teacher form. Furthermore, the excellent internal consistency should be interpreted together with the confirmatory factor analysis findings, as current psychometric guidelines emphasize that internal consistency provides meaningful evidence of reliability only after the dimensional structure of the instrument has been established (43, 44).

Although Item 24 demonstrated the lowest item–total correlation, the coefficient remained above the commonly accepted minimum threshold for item retention. Moreover, removing this item did not meaningfully improve the internal consistency of the scale. Considering its clinical relevance and contribution to the content coverage of the construct, the item was retained in the Turkish version. This decision is consistent with recent psychometric recommendations, which suggest that item retention should balance statistical performance with theoretical relevance and content coverage, rather than relying solely on internal consistency indices (44).

Item–total correlation analyses further supported each item's contribution to the overall scale, and item-deletion analyses confirmed that removing any item would not improve internal consistency, supporting retention of the original item structure (45). This pattern closely parallels the original ChAS validation study, in which all items demonstrated moderate to high item–total correlations and no item removal improved reliability, supporting preservation of the original scale structure (12). Moreover, contemporary psychometric guidance recommends preserving items that contribute to the conceptual breadth of the construct unless there is strong empirical and theoretical justification for their removal, thereby maintaining both measurement precision and content validity (44).

In addition, both the parent and teacher forms demonstrated excellent temporal stability, with high intraclass correlation coefficients (ICC = .899–.936) and no significant differences between test and retest administrations. Although the original ChAS development study established the instrument as a reliable screening tool, test–retest reliability was not evaluated (12). Therefore, the present findings extend the psychometric evidence for the ChAS-P/T by demonstrating that the Turkish versions produce stable scores over time. This level of temporal stability is consistent with recommendations for reliable screening instruments intended for repeated use in clinical and educational settings (46, 47). Furthermore, the present findings are in agreement with previous cross-cultural adaptation studies of screening questionnaires for children with motor coordination difficulties, including the French European and Turkish adaptations of the Developmental Coordination Disorder Questionnaire, which also reported excellent test–retest reliability and stable scores across repeated administrations (15, 48).

In addition, both the parent and teacher forms demonstrated excellent temporal stability, with high intraclass correlation coefficients (ICC = .899–.936) and no significant differences between test and retest administrations. Although the original ChAS development study established the instrument as a reliable screening tool, test–retest reliability was not evaluated (12). Therefore, the present findings extend the psychometric evidence for the ChAS-P/T by demonstrating that the Turkish versions produce stable scores over time. This level of temporal stability is consistent with recommendations for reliable screening instruments intended for repeated use in clinical and educational settings (46, 47). Furthermore, the excellent test–retest reliability observed in the present study may be attributable to the nature of the ChAS-P/T, which evaluates habitual and observable functional motor performance during everyday home and school activities. Because these behaviors are relatively stable over short periods in the absence of intervention or substantial developmental change, consistent scores across repeated administrations are expected when an appropriate retest interval is used (49).

Furthermore, the present findings are consistent with previous cross-cultural adaptation studies of screening instruments developed for children with motor coordination difficulties, which similarly reported excellent test–retest reliability and stable scores across repeated administrations (15, 48). Comparable findings have also been reported in pediatric activity- and participation-based outcome measures, suggesting that questionnaires assessing children's habitual functional performance in everyday environments can provide stable and reproducible measurements over time when no meaningful clinical change has occurred (50).

The consistency of the discriminant validity findings across both parent and teacher reports underscores the robustness of the Turkish ChAS-P/T across informants and indicates that the instrument captures functional difficulties manifested in both home and school environments. These findings are consistent with the original development study, in which both the ChAS-P and ChAS-T significantly distinguished children with DCD from their typically developing peers (12). The replication of this pattern in the Turkish sample provides additional evidence that the adapted forms retained the ability of the original instrument to differentiate between children with and without DCD. This discriminative performance may be explained by the ChAS-P/T's emphasis on observable difficulties in everyday functional activities rather than on isolated motor skills. Children with DCD have been shown to demonstrate significantly poorer functional performance than typically developing children across daily activities at both home and school, including self-care, mobility, fine motor activities, classroom tasks, and participation (51). Consequently, reports obtained from parents and teachers may capture complementary manifestations of motor-related functional limitations across natural contexts. This interpretation is consistent with previous literature emphasizing the importance of multi-informant questionnaires in the assessment of children with DCD (4, 13, 14).

In their comprehensive review of parent- and teacher-reported questionnaires for children with DCD, Kaiser et al. (13) noted that only a limited number of instruments demonstrated high diagnostic accuracy, particularly in terms of specificity. The ChAS-P and ChAS-T were among the instruments demonstrating specificity values of at least 90%, indicating a strong ability to correctly classify typically developing children and thereby minimize false-positive referrals. This characteristic is particularly important for a screening instrument because high specificity reduces the likelihood that children without clinically meaningful motor difficulties will be unnecessarily referred for further assessment (13). Nevertheless, screening accuracy may vary according to the characteristics of the sample, the reference standard, and the clinical context; recent meta-analytic evidence on parent-reported DCD screening questionnaires has similarly shown that diagnostic accuracy is generally stronger in clinical samples and when comprehensive diagnostic criteria are used as the reference standard (52). Therefore, the discriminant validity findings of the present study support the Turkish ChAS-P/T as a useful component of a multi-informant screening process, while its results should continue to be interpreted alongside standardized motor assessment and comprehensive clinical evaluation.

Diagnostic accuracy analyses further supported the validity of the Turkish ChAS-P/T. Receiver operating characteristic analyses demonstrated good discrimination for the parent form and excellent discrimination for the teacher form, while the optimal cut-off scores yielded favorable sensitivity and specificity values. These findings are consistent with the original development study, in which the ChAS-P/T demonstrated high specificity and was shown to be an effective screening instrument for identifying children at risk for DCD (12). The replication of these findings in the Turkish sample suggests that the cross-cultural adaptation process preserved the instrument's ability to accurately distinguish children with and without DCD.

The strong diagnostic performance of the Turkish ChAS-P/T is likely attributable to its emphasis on children's functional performance during everyday activities in natural environments rather than on isolated motor skills. Children with DCD consistently demonstrate limitations in activities of daily living, school participation, play, and other functional activities across both home and school settings, making parent- and teacher-reported questionnaires particularly suitable for identifying motor-related functional difficulties (51). This interpretation is also consistent with international clinical recommendations, which emphasize that questionnaires evaluating children's everyday functioning constitute an essential component of the diagnostic process because they capture Criterion B of the DSM-5 and complement standardized motor assessments (4).

Furthermore, the present findings are in agreement with previous evidence highlighting the value of multi-informant screening questionnaires for DCD (13, 14). Kaiser et al. (13) reported that only a limited number of parent- and teacher-reported questionnaires demonstrated high diagnostic accuracy, particularly with respect to specificity, and identified the ChAS-P and ChAS-T among the instruments with specificity values of at least 90%. Similarly, a recent systematic review and meta-analysis concluded that parent-reported DCD screening questionnaires provide acceptable diagnostic accuracy and that screening performance is generally stronger in clinically defined samples than in community-based populations (52). Collectively, these findings support the use of the Turkish ChAS-P/T as a robust multi-informant screening instrument for the early identification of children at risk for DCD, while reinforcing that questionnaire findings should be interpreted alongside standardized motor assessments and comprehensive clinical evaluation (4).

The strong positive correlations between the total and subscale scores of the ChAS-P and ChAS-T provide further evidence for the convergent validity of the Turkish versions across informants. These findings indicate that, despite observing children in different environments, parents and teachers provide largely consistent evaluations of children's functional motor performance and participation in everyday activities. The consistency of these findings with those of the original development study, which also reported a significant positive correlation between the parent and teacher forms (*r* = 0.59, *p* < .001), suggests that the Turkish adaptation preserved the complementary measurement characteristics of the original ChAS-P/T (12).

The observed agreement between parent and teacher reports is likely attributable to the design of the ChAS-P/T, which evaluates observable functional behaviors performed routinely across home and school settings rather than context-specific motor tasks. Because children with DCD typically experience persistent difficulties in activities of daily living, school participation, play, and other functional activities across multiple natural environments, similar patterns of functional limitations are expected to be recognized by both parents and teachers (4, 51). At the same time, the use of parallel parent and teacher forms enables complementary perspectives on children's functioning, providing a broader understanding of performance across contexts while reducing the influence of context-specific observations alone (4).

These findings are also consistent with contemporary evidence indicating that integrating information from multiple informants strengthens the assessment of childhood developmental conditions because different observers contribute unique yet complementary perspectives on children's everyday functioning (53). Therefore, the strong parent–teacher agreement observed in the present study enhances the practical utility of the Turkish ChAS-P/T as a multi-informant screening instrument and supports its use in informing referral decisions for comprehensive diagnostic evaluation. From an occupational therapy perspective, the Turkish ChAS-P/T provides a comprehensive evaluation of children's performance in activities of daily living, play, mobility, and school-related activities within their natural environments. By emphasizing functional performance and participation, the instrument supports occupation-based assessment, clinical reasoning, family-centered practice, and intervention planning, which are fundamental principles of the Occupational Therapy Practice Framework (OTPF-4) (54). In clinical practice, the Turkish ChAS-P/T is intended to serve as an initial screening instrument to identify children who may require comprehensive assessment rather than as a stand-alone diagnostic measure. The complementary parent and teacher forms enable occupational therapists to compare children's functioning across home and school contexts, facilitating interdisciplinary communication among families, teachers, and healthcare professionals while supporting family-centered decision-making and participation-focused goal setting. Information obtained from the ChAS-P/T may also assist therapists in identifying priority areas for further assessment and occupation-based intervention planning.

These clinical implications are further strengthened by the methodological rigor of the present study. A major strength of this study is the comprehensive evaluation of the psychometric properties of the Turkish versions of the ChAS-P and ChAS-T using a multi-informant approach. The inclusion of both parent and teacher reports enabled the assessment of children's functional performance across home and school contexts, providing a broader understanding of everyday participation. Furthermore, the study employed a relatively large sample and multiple complementary psychometric methods, including confirmatory factor analysis, internal consistency, test–retest reliability, known-groups comparisons, and receiver operating characteristic analyses. Together, these findings provide robust evidence supporting the reliability, validity, and diagnostic accuracy of the Turkish ChAS-P/T as a screening instrument for identifying preschool-aged children at risk for developmental coordination disorder.

Despite these strengths, several limitations should be acknowledged. First, participants were recruited from a single metropolitan area, which may limit the generalizability of the findings to children from other regions of Türkiye with different cultural and sociodemographic characteristics. Second, the clinical DCD group was relatively small (*n* = 34), and recruitment from occupational therapy clinics may have introduced spectrum bias, potentially limiting the representativeness of the clinical sample. Third, the cross-sectional design precludes conclusions regarding the responsiveness of the Turkish ChAS-P/T or its suitability for monitoring changes in functional performance over time. Furthermore, several additional psychometric properties, including measurement error indices (e.g., SEM and MDC), floor and ceiling effects, and measurement invariance, were not evaluated because they were beyond the scope of the present study. Finally, although diagnostic accuracy was examined, children were not evaluated using a standardized performance-based motor assessment or a comprehensive clinical diagnostic procedure. Future studies should include larger and more geographically diverse samples, clinically confirmed populations, longitudinal designs, and additional psychometric analyses to further strengthen the evidence supporting the Turkish ChAS-P and ChAS-T and their clinical applicability.

## 5 Conclusions

This study translated and culturally adapted the ChAS-P/T into Turkish and provided comprehensive evidence supporting their psychometric properties in children aged 4–8 years. The findings indicate that the Turkish versions retain the original conceptual and structural framework of the instrument while demonstrating satisfactory validity and strong reliability across both parent and teacher forms. These results support the cross-cultural applicability of the ChAS-P/T and suggest that the Turkish versions provide a consistent and meaningful assessment of children's functional motor performance in home and school environments. The Turkish ChAS-P/T may support occupational therapists and other healthcare professionals in the early identification and functional evaluation of children at risk for developmental coordination disorder, facilitating occupation-based assessment, intervention planning, and multidisciplinary clinical decision-making. Furthermore, the instrument may serve as a valuable outcome measure in educational settings and future research involving Turkish-speaking populations.

## Data Availability

The datasets generated and analyzed during the current study are not publicly available due to ethical and privacy restrictions involving human participants but are available from the corresponding author on reasonable request. Requests to access the datasets should be directed to: Duygu Demirbaş Email: duygudemirbaass@gmail.com.
